# Utility of Anthropometric Indexes for Detecting Metabolic Syndrome in Resource-Limited Regions of Northwestern China: Cross-Sectional Study

**DOI:** 10.2196/57799

**Published:** 2024-11-29

**Authors:** Danyu Yang, Ling Ma, Yin Cheng, Hongjuan Shi, Yining Liu, Chao Shi

**Affiliations:** 1 People’s Hospital of Ningxia Hui Autonomous Region, Ningxia Medical University, No.301 Zhengyuan North Street, Yinchuan, 750001, China, 86 10-5920152, 86 10-5920017; 2 School of Public Health, Ningxia Medical University, Yinchuan, China

**Keywords:** metabolic syndrome, MetS, anthropometric indexes, lipid accumulation product, LAP, waist-to-height ratio, WHtR, anthropometric, adult, aging, NingXia, China, cross-sectional study, population-based survey, logistic regression, waist-to-height, threshold, diagnosis, public health

## Abstract

**Background:**

Anthropometric indexes offer a practical approach to identifying metabolic syndrome (MetS) and its components. However, there is a scarcity of research on anthropometric indexes tailored to predict MetS in populations from resource-limited regions.

**Objective:**

This study aimed to examine the association between 8 easy-to-collect anthropometric indexes and MetS, and determine the most appropriate indexes to identify the presence of MetS for adults in resource-limited areas.

**Methods:**

A total of 10,520 participants aged 18‐85 years from Ningxia Hui Autonomous Region, China, were included in this cross-sectional study. Participants were recruited through a stratified sampling approach from January 1, 2020, to December 31, 2021. MetS was defined using the International Diabetes Federation (IDF) criteria. Eight anthropometric indexes were examined, including BMI, waist-to-height ratio (WHtR), weight-adjusted waist index (WWI), conicity index, a body shape index (ABSI), lipid accumulation products (LAP), visceral obesity index (VAI), and the triglyceride-glucose (TyG) index. Logistic regression analysis and restricted cubic splines (RCSs) were applied to identify the association between the anthropometric indexes. The receiver operating characteristic curve and the area under the curve (AUC) were analyzed to identify and compare the discriminative power of anthropometric indexes in identifying MetS. The Youden index was used to determine a range of optimal diagnostic thresholds. Logistic regression analysis was applied to identify the association between the anthropometric indexes.

**Results:**

A total of 3324 (31.60%) participants were diagnosed with MetS. After adjusting for age, ethnicity, current residence, education level, habitual alcohol consumption, and tobacco use, all the 8 indexes were positively correlated with the risks of MetS (*P*<.05). LAP presented the highest adjusted odds ratios (adjOR 35.69, 95% CI 34.59‐36.80), followed by WHtR (adjOR 29.27, 95% CI 28.00‐30.55), conicity index (adjOR 11.58, 95% CI 10.95‐12.22), TyG index (adjOR 5.53, 95% CI 5.07‐6.04), BMI (adjOR 3.88, 95% CI 3.71‐4.05), WWI (adjOR 3.23, 95% CI 3.02‐3.46), VAI (adjOR 2.11, 95% CI 2.02‐2.20), and ABSI (adjOR 1.71, 95% CI 1.62‐1.80). Significantly nonlinear associations between the 8 indexes and the risk of MetS (all *P*_nonlinear_<.001) were observed in the RCSs. WHtR was the strongest predictor of MetS for males (AUC 0.91, 95% CI 0.90-0.92; optimal cutoff 0.53). LAP were the strongest predictor of MetS for females (AUC 0.89, 95% CI 0.89-0.90; optimal cutoff 28.67). Statistical differences were present between WHtR and all other 7 anthropometric indexes among males and overall (all *P*<.05). In females, the AUC values between LAP and BMI, WWI, ABSI, conicity index, VAI, and TyG index were significantly different (*P*<.001). No statistical difference was observed between LAP and WHtR among females.

**Conclusions:**

According to 8 anthropometric and lipid-related indices, it is suggested that WHtR and LAP are the most appropriate indexes for identifying the presence of MetS in resource-limited areas.

## Introduction

Metabolic syndrome (MetS) is a group of interrelated metabolic abnormalities that include overweight and abdominal fat accumulation, moderate dyslipidemia, hypertension, and impaired glucose metabolism [1,2]. It has been identified and spotlighted for its close association with diabetes, cardiovascular complications, and all-cause mortality as well as for its widespread prevalence in resource-limited regions [3-6]. People living in these regions are more likely to experience the trend toward energy-dense, nutrient-poor dietary patterns and sedentary lifestyles due to rapid urbanization [4]. Despite extensive investigations of MetS, studies focusing on its early detection strategies and predictive measures in different populations have been uneven, with the resource-limited areas of China being particularly underrepresented. These resource-limited areas have been observed to experience larger scales and higher rates of urbanization than traditional development trajectories [7], highlighting the need for conducting region-specific validation studies to effectively identify MetS.

Anthropometric indexes offer a practical approach to identifying MetS and its components [8]. In terms of clinical assessment, anthropometric indexes provide evidence of individuals’ historical and current physical health and nutritional status [9]. They are also practical to monitor malnutrition and changes in body composition [9]. Previous studies have highlighted straightforward collection and interpretation, standardized measuring, and noninvasiveness as the advantages of anthropometric indexes in health screening [10-12]. These indexes are critical for identifying at-risk individuals in clinical and community settings in resource-limited areas with limited health care resources [13].

The limitations of predicting MetS by relying solely on height and weight variables to assess body fat distribution among Chinese populations have been reported in several studies [12,14-16]. These studies suggested applying alternative simple measures to compensate for this limitation [12,15,16]. However, the participants in these studies were either limited to specific age groups or recruited from hospitals or health care centers [12,14], which may limit the representativeness of their findings to the general population. Besides, indexes examined in some of these studies were only based on body measurements, while indexes referring to plasma lipid profiles and glycemic status were overlooked. Blood profile indexes have been found to provide better monitoring of chronic disease progression in clinical settings, such as diabetes and cardiovascular diseases (CVDs) [9]. Therefore, incorporating blood profile indices into MetS research is suggested.

To date, there is a scarcity of research on anthropometric indexes tailored to identify the presence of MetS in populations from resource-limited areas of China. To fill these gaps, this study aimed to explore the relationship between 8 anthropometric indexes, including both body measurements and blood profiles, and the risk of MetS in adults from resource-limited areas. It also aims to compare their predictive powers to determine the most appropriate simple tool for MetS screening. The findings of this research would provide evidence to optimize health care prevention strategies against metabolic abnormalities, particularly in resource-limited areas. These findings would also contribute to the development of cost-effective, accessible early screening programs by using the most predictive anthropometric indexes, thereby facilitating early detection and management of MetS in communities with limited health care resources.

## Methods

### Study Population

Ningxia Hui Autonomous Region was defined as one of the most resource-limited inland areas in Northwest China by the World Bank, with more than 50% of its counties classified as poverty counties [17]. From January 1, 2020, to December 31, 2021, a population-based survey was conducted in this region to investigate the prevalence and risk factors of CVDs, including coronary heart disease, obesity, hypertension, dyslipidemia, diabetes mellitus, and hyperuricemia (Ningxia cardiovascular disorders and the related risk factors survey). This study used the samples from this survey. The design of the Ningxia cardiovascular disorders and the related risk factors survey has been described elsewhere [18]. Briefly, a 4-stage, stratified cluster sampling method was used to select a regionally representative sample of the general population aged 18 years and older. In the first stage, 9 counties with varied socioeconomic statuses that represent both urban and rural areas were chosen as survey sites. The selected counties included Yinchuan, Wuzhong, Zhongwei, Guyuan, and Shizuishan (Figure S1 in Multimedia Appendix 1). In the second stage, 2 towns in each county were selected using simple random sampling according to the list of towns provided by the local Center for Disease Control and Prevention. In the third stage, 3 communities or villages in each selected town were selected using Simple Random Sampling. The final stage of sampling was stratified by sex and age distribution according to the China census data from 2010. A total of 10,803 permanent residents were recruited as study participants.

All participants in this study were required to meet the following criteria: (1) adults between 18 and 85 years old and (2) having signed consent to participate in this study and associated assessments. Participants were dropped from the study if they met any of these conditions: (1) participants with incomplete demographic, anthropometric, or laboratory data; (2) having cancer or end-stage kidney disease; and (3) receiving surgical treatments within 6 months before sampling. The total eligible participants for this study were 10,520 (Figure S2 in Multimedia Appendix 1).

### Anthropometric Data Measurements

Face-to-face, interviewer-administered surveys were conducted to collect the demographic characteristics and lifestyle factors of participants. Anthropometric indexes were measured using standard procedures by well-trained health care professionals at community or village health care centers. Anthropometric data including body weight, muscle mass, fat mass, visceral fat level, and body fat percentage, were measured by a smart body scale (InBody H20N) under a standardized procedure. A nonelasticated measuring tape was used to measure waist circumference (WC). The accuracy of this tape was 1 millimeter (mm). The tape was placed at the midpoint between the inferior edge of the last rib and the superior edge of the iliac crest. It was then horizontally wrapped around the waist at the end of a normal exhalation, with the participants standing naturally and legs positioned approximately 30‐40 cm apart. Participants’ standing height was measured by a stadiometer with an accuracy of 1 mm under a standardized procedure. After a 5-minute rest, an electronic sphygmomanometer (OMRON, HBP-1120U) was used to measure blood pressure in the right arm in a sitting position 3 times and the average of these readings was collected.

### Laboratory Data Measurements

Participants were required to conduct an overnight fasting before the blood test (at least 8 hours but not more than 16 hours). Approximately 5 mL of venous blood samples from participants were collected and processed at the People’s Hospital of Ningxia Hui Autonomous Region. These samples were stored at −80 ℃, packaged in dry ice, and transported altogether to the Beijing CIC Medical Laboratory for analysis. An AU5800 Chemistry Analyzer (Beckman Coulter, California, USA) was used to measure the level of total cholesterol (TC), serum low-density lipoprotein cholesterol (LDL-C), serum high-density lipoprotein cholesterol (HDL-C), serum triglycerides (TG), fasting plasma glucose (FPG), serum uric acid, and serum creatinine. Commercial reagents (Biosino, Beijing, China) were used in this measuring process. The Tosoh Automated Glycohemoglobin Analyzer HLC-723GX (Tosoh Corporation, Tokyo, Japan) was used to measure the level of glycated hemoglobin (HbA_1c_). All laboratory practices adhered to standardized procedures.

### Anthropometric Indexes Calculation and MetS Definition

In this study, BMI, waist-to-height ratio (WHtR), weight-adjusted waist index (WWI), conicity index, a body shape index (ABSI), lipid accumulation products (LAP), visceral obesity index (VAI), and the triglyceride-glucose (TyG) index were considered to predict the presence of MetS. These 8 indexes are calculated as follows:

- BMI = body weight (kg)/height (m)^2^ [19]
- WHtR = WC (cm)/height (cm) [20]
- WWI = $\frac{WC\;\left(cm\right)}{\sqrt{body\;weight\;\left(kg\right)}}$ [21]
- Conicity index = WC (m)/(0.109 × $\sqrt{\frac{body\;weight\;\left(kg\right)}{height\;\left(m\right)}}$) [22]
- ABSI = WC (m)/[BMI^2/3^ (kg/m^2^) × height^1/2^ (m)] [23]
- LAP (female) = [WC (cm) – 58] × TG (mmol/L)
- LAP (male) = [WC (cm) – 65] × TG (mmol/L) [24]
- VAI (female) = $\frac{WC\;\left(cm\right)}{36.58\;+\;\left[1.89\;\times \;BMI\left(kg/m^{2}\right)\right]}$ × $\frac{TG\;\left(mmol/L\right)}{0.81}\;\times \;\frac{1.52}{HDL-C\;\left(mmol/L\right)}$
- VAI (male) = $\frac{WC\;\left(cm\right)}{39.68\;+\;\left[1.88\;\times \;BMI\left(kg/m^{2}\right)\right]}$ × $\frac{TG\;\left(mmol/L\right)}{1.03}\;\times \;\frac{1.31}{HDL-C\;\left(mmol/L\right)}$ [25]
- TyG index = ln × FPG (mg/dL)/2] [26]

According to the International Diabetes Federation (IDF), MetS is defined as central obesity (ie, WC≥90 cm for males and ≥80 cm for females), combined with any two of these four risk factors: (1) elevated TG≥1.7 mmol/L or under relevant treatments; (2) reduced HDL-C<1.29 mmol/L for females and <1.03 mmol/L for males or under relevant treatments; (3) systolic blood pressure (SBP)≥130 mm Hg or diastolic blood pressure (DBP)≥85 mm Hg or under relevant treatments; and (4) raised FPG≥5.6 mmol/L or was diagnosed with diabetes previously [2]. FPG≥7.0 mmol/L and HbA_1c_≥6.5% were suggested for people with diabetes instead of FPG levels solely [2].

### Ethical Considerations

This study received ethical approval from the Institutional Review Board of the People’s Hospital of Ningxia Hui Autonomous (2020-YC-002). All participants provided informed consent. All participants provided informed consent, and no compensation was offered to them. During the data analysis process, all participants' names, identification numbers, and phone numbers were anonymized.

### Statistical Analyses

All analyses were conducted through the STATA MP17 (StataCorp). Descriptive statistics were used to describe participants’ anthropometric and laboratory data measurements. Continuous data with approximately normal distributions were described as mean (SD), and those with skewed distributions were described as median (IQR). The normality was assessed using the Kolmogorov-Smirnov test. The characteristic differences between the non-MetS group and MetS group were examined using the chi-square test and *z*-test for categorical and continuous variables, respectively. Logistic regression analysis and restricted cubic splines (RCSs) were applied to identify the association between the anthropometric indexes (BMI, WHtR, WWI, conicity index, ABSI, LAP, VAI, TyG index) and the presence of MetS. The receiver operating characteristic curve and the area under the curve (AUC) were analyzed to identify and compare the discriminative power of anthropometric indexes in identifying MetS. The Youden index was used to determine a range of optimal diagnostic thresholds. *P* values <.05 were used to characterize statistically significant results.

## Results

### Baseline Characteristics of the Participants

**Figure 1.**
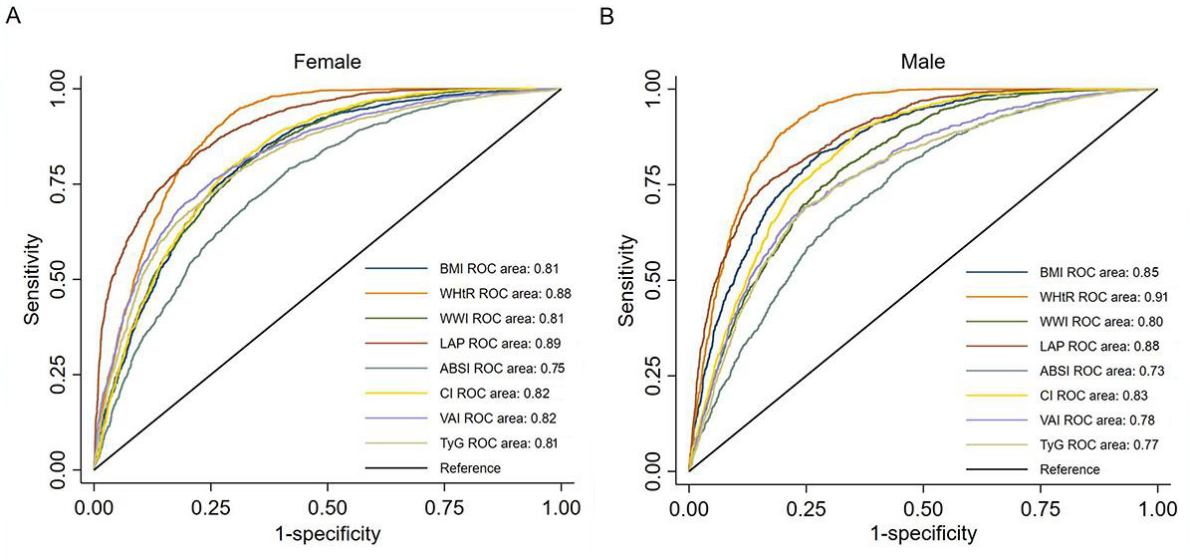
The values of 8 anthropometric indexes according to the number of metabolic syndrome components in both sexes among 10,520 individuals from Ningxia Hui Autonomous Region, 2020-2021. ABSI: a body shape index; CI: conicity index; LAP: lipid accumulation products; ROC: receiver operating characteristic; TyG index: triglyceride-glucose index; VAI: visceral obesity index; WHtR: waist-to-height ratio; WWI: weight-adjusted waist index.

Participants’ baseline characteristics are summarized in Table 1. A total of 3324 (31.60%) participants were diagnosed with MetS, with 1905 (57.31%) of them being females and 1419 (42.69%) being males. When compared with the non-MetS group, the MetS group was older, was mostly female, and had a lower educational level (*P*<.001). This group also had a significantly higher fat mass, visceral fat level, and body fat percentage (all *P*<.001) and lower muscle mass (*P*<.001) than the non-MetS group. For the antropometric data, the MetS group had significantly higher levels of BMI, WHtR, WWI, LAP, VAI, and TyG index (*P*<.001). For laboratory outcomes, significantly increased levels of HbA_1c_, FPG, TG, TC, LDL-C, SBP, and DBP (all *P*<.001) and significantly lower levels of HDL-C (*P*<.001) were observed in the MetS group. The values of BMI, WHtR, WWI, conicity index, ABSI, LAP, VAI, and TyG index increased with the number of MetS risk factors (Figure 1). No statistical difference between sexes was observed in all numbers of risk factors for WHtR. Statistically significant differences in BMI, WWI, and conicity index between sexes were observed for participants with more than 3 MetS risk factors (*P*<.001).

**Table 1.** General characteristics of the sample participants stratified by the presence of MetS^a^ in 10,520 individuals from Ningxia Hui Autonomous Region, 2020‐2021.

Characteristics	Overall (n=10,520)	Non-MetS (n=7196)	MetS (n=3324)	*P* value
Age (years), median (IQR)	46 (33, 59)	42 (30, 55)	54 (43, 67)	<.001^b^
Sex, n (%)				<.001^c^
Female	5679 (53.98)	3774 (52.45)	1905 (57.31)	
Male	4841 (46.02)	3422 (47.55)	1419 (42.69)	
Ethnicity, n (%)				.55^c^
Han	7460 (70.91)	5094 (70.79)	2366 (71.18)	
Hui	2864 (27.22)	1961 (27.25)	903 (27.17)	
Others	196 (1.86)	141 (1.98)	55 (1.65)	
Habitual alcohol consumption^d^, n (%)				.03^c^
Yes	2227 (21.17)	1567 (70.36)	660 (29.64)	
No	8293 (78.83)	5629 (67.88)	2664 (32.12)	
Habitual tobacco use^e^, n (%)				.16^c^
Yes	2517 (23.93)	1750 (69.53)	767 (30.47)	
No	8002 (76.07)	5445 (68.05)	2557 (31.95)	
Education level, n (%)				<.001^c^
Primary school or below	3707 (35.24)	2074 (28.82)	1633 (49.13)	
Middle school	3016 (28.67)	2015 (28.00)	1001 (30.11)	
High school or above	3797 (36.09)	3107 (43.18)	690 (20.76)	
Anthropometry data, mean (SD)				
Weight (kg)	66.46 (12.58)	63.16 (10.97)	73.59 (12.90)	<.001^f^
Height (cm)	162.65 (8.74)	162.98 (8.51)	161.93 (9.18)	<.001^f^
BMI (kg/m^2^)	25.05 (3.87)	23.72 (3.35)	27.93 (3.32)	<.001^f^
Waist circumference (cm)	83.65 (11.70)	79.00 (9.90)	93.73 (8.49)	<.001^f^
Muscle mass (kg)	24.55 (5.42)	24.08 (5.07)	25.56 (5.98)	<.001^f^
Fat mass (kg)	21.73 (7.38)	19.25 (6.43)	27.12 (6.37)	<.001^f^
Visceral fat level (level)	10.22 (4.20)	8.77 (3.67)	13.36 (3.50)	<.001^f^
Body fat percentage (%)	32.32 (7.88)	30.19 (7.64)	36.93 (6.25)	<.001^f^
WHtR^g^	0.52 (0.73)	0.49 (0.06)	0.58 (0.05)	<.001^f^
WWI^h^	10.30 (1.02)	9.97 (0.90)	11.00 (0.92)	<.001^f^
LAP^i^	37.23 (42.86)	22.49 (22.49)	69.15 (56.83)	<.001^f^
ABSI^j^	0.08 (0.01)	0.08 (0.02)	0.08 (0.01)	.80^f^
Conicity index	1.20 (0.11)	1.17 (0.01)	1.28 (0.01)	<.001^f^
VAI^k^	2.02 (2.25)	1.49 (1.49)	3.17 (3.05)	<.001^f^
TyG^l^ index	7.00 (0.70)	6.79 (0.62)	7.46 (0.66)	<.001^f^
Laboratory data, mean (SD)				
HbA_1c_^m^ (%)	5.64 (0.78)	5.49 (0.62)	5.96 (0.98)	<.001^f^
FPG^n^ (mmol/L)	5.71 (1.46)	5.42 (1.15)	6.32 (1.87)	<.001^f^
TG^o^ (mmol/L)	1.50 (1.33)	1.22 (1.04)	2.10 (1.64)	<.001^f^
TC^p^ (mmol/L)	4.27 (0.97)	4.17 (0.91)	4.50 (1.05)	<.001^f^
HDL-C^q^ (mmol/L)	1.26 (0.28)	1.31 (0.28)	1.16 (0.24)	<.001^f^
LDL-C^r^ (mmol/L)	2.59 (0.78)	2.51 (0.75)	2.77 (0.81)	<.001^f^
SBP^s^ (mm Hg)	130.32 (19.96)	124.74 (18.05)	142.39 (18.53)	<.001^f^
DBP^t^ (mm Hg)	81.03 (11.16)	78.53 (10.44)	86.46 (10.74)	<.001^f^
MetS components				
Elevated waist circumference, n (%)				<.001^c^
Yes	4966 (47.21)	1642 (22.82)	3324 (100.00)	
No	5554 (52.79)	5554 (77.18)	0 (0.00)	
Elevated TG, n (%)				<.001^c^
Yes	2937 (27.92)	1139 (15.83)	1798 (54.09)	
No	7583 (72.08)	6057 (84.17)	1526 (45.91)	
Elevated blood pressure, n (%)				<.001^c^
Yes	5350 (50.86)	2594 (36.05)	2756 (82.91)	
No	5170 (49.14)	4602 (63.95)	568 (17.09)	
Reduced HDL-C level, n (%)				<.001^c^
Yes	6428 (61.10)	2118 (29.43)	1974 (59.39)	
No	4092 (38.90)	5078 (70.57)	1350 (40.61)	
Raised FPG, n (%)				<.001^c^
Yes	4382 (41.65)	2068 (28.74)	2314 (69.61)	
No	6138 (58.35)	5128 (71.26)	1010 (30.39)	

a MetS: metabolic syndrome.

b *P* value was obtained from Kruskal-Wallis test.

c *P* values were obtained from chi-square test.

d Participants who reported “at least once a day” in response to the drinking habits question in the survey were classified as habitually consuming alcohol*.*

e Participants who reported “at least once a day” in response to the tobacco use habits question in the survey were classified as habitually using tobacco.

f *P* values were obtained from Z test.

g WHtR: waist-to-height ratio.

h WWI: weight-adjusted waist index.

i LAP: lipid accumulation products.

j ABSI: a body shape index.

k VAI: visceral obesity index.

l TyG index: triglyceride-glucose index.

m HbA_1c_: glycated hemoglobin.

n FPG: fasting plasma glucose.

o TG: triglycerides.

p TC: total cholesterol.

q HDL-C: high density lipoprotein cholesterol.

r LDL-C: low density lipoprotein cholesterol.

s SBP: systolic blood pressure.

t DBP: diastolic blood pressure.

### Associations Between 8 Anthropometric Indexes and the Risk of MetS

The multivariate-adjusted odds ratios (ORs) and 95% CIs for the risks of MetS in relation to the levels of the 8 predictive indexes are summarized in Table 2. After adjusting for age, ethnicity, current residence, education level, habitual alcohol consumption, and tobacco use, all BMI, WHtR, WWI, conicity index, ABSI, LAP, VAI, and TyG index values were positively correlated with the risks of MetS. LAP presented the highest OR (OR 35.69, 95% CI 34.59‐36.80), followed by WHtR (OR 29.27, 95% CI 28.00‐30.55), conicity index (OR 11.58, 95% CI 10.95‐12.22), TyG index (OR 5.53, 95% CI 5.07‐6.04), BMI (OR 3.88, 95% CI 3.71‐4.05), WWI (OR 3.23, 95% CI 3.02‐3.46), VAI (OR 2.11, 95% CI 2.02‐2.20), and ABSI (OR 1.71, 95% CI 1.62‐1.80). Significantly nonlinear associations between BMI, WHtR, WWI, conicity index, ABSI, LAP, VAI, and TyG index and the risk of MetS (all *P*_nonlinear_<.001) were observed in the RCSs (Figure S3 in Multimedia Appendix 1). Differences were observed in the distribution profiles of participants and the slope of curves in the RCSs between females and males.

**Table 2.** Multivariate adjusted ORs^a^ of metabolic syndrome in relation to 8 predictive indexes in 10,520 individuals from Ningxia Hui Autonomous Region, 2020‐2021. Adjusted for age (years), ethnicity, current residence, education level, habitual alcohol consumption, and tobacco use.

Index	Crude OR (95% CI)	*P* values	Adjusted OR (95 CI%)	*P* values
BMI	4.06 (3.89‐4.22)	<.001	3.88 (3.71‐4.05)	<.001
WHtR^b^	30.02 (28.79‐31.25)	<.001	29.27 (28.00‐30.55)	<.001
WWI^c^	3.69 (3.47‐3.93)	<.001	3.23 (3.02‐3.46)	<.001
LAP^d^	37.74 (36.67‐38.81)	<.001	35.69 (34.59‐36.80)	<.001
ABSI^e^	1.68 (1.60‐1.76)	<.001	1.71 (1.62‐1.80)	<.001
Conicity index	12.28 (11.70‐12.86)	<.001	11.58 (10.95‐12.22)	<.001
VAI^f^	2.14 (2.05‐2.22)	<.001	2.11 (2.02‐2.20)	<.001
TyG^g^ index	5.37 (4.95‐5.83)	<.001	5.53 (5.07‐6.04)	<.001

a OR: odds ratio.

b WHtR: waist-to-height ratio.

c WWI: weight-adjusted waist index.

d LAP: lipid accumulation products.

e ABSI: a body shape index.

f VAI: visceral obesity index.

g TyG index: triglyceride-glucose index.

### Discrimination of 8 Anthropometric Indexes for Identifying the Presence of MetS

**Figure 2.**
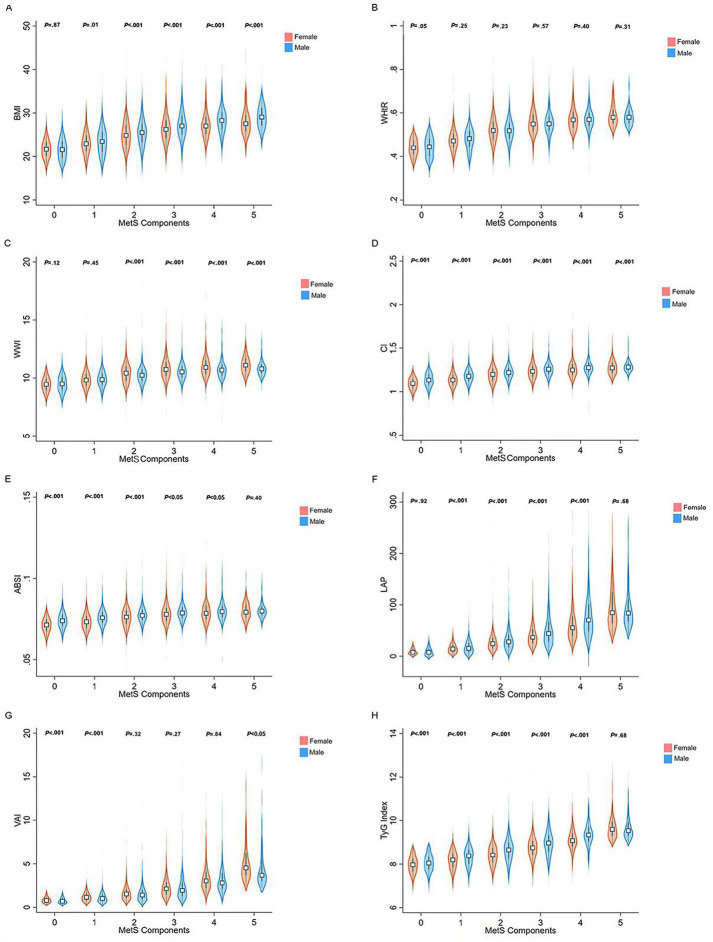
Area under the receiver operating curves of different anthropometric indexes to identify the presence of MetS in 10,520 individuals from Ningxia Hui Autonomous Region, 2020-2021. ABSI: a body shape index; CI: conicity index; LAP: lipid accumulation products; MetS: metabolic syndrome; TyG index: triglyceride-glucose index; VAI: visceral obesity index; WHtR: waist-to-height ratio; WWI: weight-adjusted waist index.

Figure 2 presents the AUC values for anthropometric indexes to identify the presence of MetS. WHtR showed the highest AUC for males (0.91, 95% CI 0.90‐0.92). LAP showed the highest AUC for females (0.89, 95% CI 0.89‐0.90). The optimal cutoff value and its corresponding sensitivity, specificity, and Youden index can be found in Table S1 in Multimedia Appendix 1. After stratifying by age, WHtR and LAP also had the highest AUC values among all age groups (Table S2 in Multimedia Appendix 1). The AUC values of all 8 anthropometric indexes declined with age.

### Comparison of AUC Values Between 8 Anthropometric Indexes in Identifying the Presence of MetS

The comparisons of AUC values between BMI, WHtR, WWI, conicity index, ABSI, LAP, VAI, and TyG index to predict MetS are shown in Table 3. Statistical differences were present between WHtR and all other 7 anthropometric indexes among males and overall (all *P*<.05). In females, the AUC values between LAP and BMI, WWI, ABSI, conicity index, VAI, and TyG index were significantly different (*P*<.001). No statistical difference was observed between LAP and WHtR among females.

**Table 3.** Comparison of area under the curve (AUC) values between 8 anthropometric indexes in identify the presence of metabolic syndrome in 10,520 individuals from Ningxia Hui Autonomous Region, 2020‐2021.

Sex and index (AUC value)		*P* values^a^							
		BMI	WHtR^b^	WWI^c^	LAP^d^	ABSI^e^	Conicity index	VAI^f^	TyG^g^ Index
Female									
	BMI (AUC=0.81)	—^h^	<.001	.81	<.001	<.001	.26	.08	.86
	WHtR (AUC=0.88)	—	—	<.001	.06	<.001	<.001	<.001	<.001
	WWI (AUC=0.81)	—	—	—	<.001	<.001	<.001	.13	.69
	LAP (AUC=0.89)	—	—	—	—	<.001	<.001	<.001	<.001
	ABSI (AUC=0.75)	—	—	—	—	—	<.001	<.001	<.001
	Conicity index (AUC=0.82)	—	—	—	—	—	—	.56	.20
	VAI (AUC=0.82)	—	—	—	—	—	—	—	<.001
	TyG index (AUC=0.81)	—	—	—	—	—	—	—	—
Male									
	BMI (AUC=0.85)	—	<.001	<.001	<.001	<.001	.08	<.001	<.001
	WHtR (AUC=0.91)	—	—	<.001	<.001	<.001	<.001	<.001	<.001
	WWI (AUC=0.80)	—	—	—	<.001	<.001	<.001	<.001	<.001
	LAP (AUC=0.88)	—	—	—	—	<.001	<.001	<.001	<.001
	ABSI (AUC=0.73)	—	—	—	—	—	<.001	<.001	<.001
	Conicity index (AUC=0.83)	—	—	—	—	—	—	<.001	<.001
	VAI (AUC=0.78)	—	—	—	—	—	—	—	<.001
	TyG index (AUC=0.77)	—	—	—	—	—	—	—	—

a *P* values were derived from the comparison of AUC values between 8 anthropometric indexes in identifying the presence of metabolic syndrome.

b WHtR: waist-to-height ratio.

c WWI: weight-adjusted waist index.

d LAP: lipid accumulation products.

e ABSI: a body shape index.

f VAI: visceral obesity index.

g TyG index: triglyceride-glucose index.

h Not applicable.

In addition, the TyG index presented the highest AUC values for early identifications of elevated TG (0.98, 95% CI 0.98‐0.98) and raised FPG (0.72, 95% CI 0.71‐0.73), with optimal cutoff values of 9.00 and 8.65, respectively (Table S3 in Multimedia Appendix 1). VAI and LAP also showed high AUC values for elevated TG. The optimal cutoff values for VAI and LAP to early identify elevated TG were 2.63 (AUC=0.97, 95% CI 0.96‐0.97) and 55.33 (AUC=0.93, 95% CI 0.93‐0.94), respectively.

## Discussion

### Principal Findings

In this cross-sectional study, we explored the associations between 8 easy-to-collect anthropometric indexes (BMI, WHtR, WWI, conicity index, ABSI, LAP, VAI, and TyG index) and the risk of MetS, and also assessed their diagnostic performances to identify the presence of MetS in resource-limited areas of China. We found that WHtR and LAP were more influential risk factors for developing MetS than the traditional index BMI. Both of them had the most superior abilities to predict MetS, suggesting high applicability in future clinical screening programs. However, the diagnostic performances of these indexes varied by sex. It seems indexes referring to plasma lipid profiles had a greater ability to predict MetS in females. Besides, the diagnostic power of these indexes diminished with age. Anthropometric indexes, regardless of being body measurements or blood profile indexes, are less effective in accounting for age-related changes. More comprehensive metabolic assessments are required for older populations to identify MetS risk.

The prevalence of MetS in this resource-limited region was 31.60% under the IDF definition, which is higher than the overall prevalence in the Southeast Asia Region (28.1%) [5]. This greater incidence is closely associated with the growth of processed food industries as a consequence of rapid urbanization, which has increased the accessibility and consumption of ultraprocessed foods high in fat and sugar among all ages of the population [27]. Higher intake of ultraprocessed foods was found to increase the risk of visceral and overall adiposity accumulation, with both of them being primary risk factors for MetS and CVD comorbidities [28].

In our study, WHtR demonstrated the greatest diagnostic accuracy in predicting MetS under the IDF criteria. Similar results were observed in previous studies that WHtR had stronger abilities to identify MetS among the Vietnamese and Korean populations [29,30]. WHtR does have the capacity to compensate for the disadvantage of indexes relying solely on height and weight variables in assessing body fat distribution when identifying MetS. The optimal thresholds of WHtR were 0.51 for females and 0.53 for males to predict MetS in our study. The same thresholds were observed among Vietnamese communities, with 0.51 for females and 0.53 for males, but higher thresholds are suggested for Polish populations with 0.54 for females and 0.56 for males [29,31]. In another cross-sectional study conducted among Thai adolescents, the optimal thresholds for WHtR to diagnose MetS were found to be 0.49 for females and 0.51 for males [32]. In addition to its greater diagnostic power, the measurement and calculation of WHtR are more straightforward than WWI, conicity index, and ABSI. Based on the findings of a recent study, the perception of health screening procedures as too complex to understand is a key barrier for residents to participate in chronic disease management [33]. By simplifying the measurement process, WHtR is potent to encourage more residents in resource-limited areas to engage in regular MetS screening and associated early interventions, therefore narrowing health care disparities between resource-abundant and resource-limited areas.

As the most effective diagnostic index in females, LAP are a novel index that provides information on individuals’ visceral fat based on circulating TG levels and WC [24]. Visceral fat deposition is the main pathogenesis of MetS [10]. When compared with subcutaneous adipose tissue, visceral adipose tissue was found to have higher lipolytic activity and then a greater contribution to free fatty acid levels in the body circulation [34]. Therefore, it is important to include a reliable blood index to assess visceral fat routinely to further support WHtR in screening individuals without obvious physical symptoms of MetS but presenting with metabolic abnormalities. Fat deposition in females is usually about 10% higher than in males at the same BMI [35], which likely contributes to the superior predictive performance of LAP in females than in males. From a comparative perspective, LAP had a greater diagnostic value among females and lesser among males, but its performance was consistent between both sexes. The optimal thresholds of LAP were 28.67 for females and 40.23 for males to predict MetS in our study. The female LAP cutoff is close to the value found in a recent study conducted among Korean adults, which suggested a cutoff of 25.71 for the metabolically unhealthy nonobese female population, whereas the male LAP cutoff is close to the value of 41.45 for the metabolically unhealthy obese male population [36]. The presence of different obesity phenotypes in our female and male participants is likely to explain the distinct disparities between sexes in the suggested optimal LAP cutoffs for diagnosing MetS. Higher cutoffs were suggested for the young Indian population where the thresholds are 46.91 for females and 45.65 for males, and for the US population where the thresholds are 52.43 for females and 53.31 for males [37,38].

Surprisingly, as an important blood marker for insulin resistance and CVD [26], the TyG index did not show a stronger predictive power in our study, particularly in those older than 35 years. We speculate this may be related to the presence of factors that cause FPG fluctuations such as age-related alteration of insulin sensitivity and pancreatic function. MetS is a group of interactive and progressive metabolic abnormalities [2]. The development of associated disorders such as pancreatitis induced by hypertriglyceridemia may result in stress hyperglycemia (ie, rapid rise of FPG in a short period), which degrades the predictive power of the TyG index. According to the IDF definition of MetS, participants with “previous diagnosis of T2DM” [2] were included in our study. Tao et al [26] highlighted that the symptoms of diabetes might interfere with the application of the TyG index. This could be the main reason that findings from a study conducted on nondiabetic individuals showed a higher AUC value for the TyG index (0.877, 95% CI 0.814‐0.940) than our study (0.79, 95% CI 0.78‐0.80) [37]. The effectiveness of the TyG index for identifying the presence of MetS was also found to be higher when using the harmonized criteria than the IDF criteria [37]. Therefore, the diagnostic power of the TyG index seems to be limited in populations with limited control of glucose metabolic disorders or by the use of the IDF criteria.

This is one of the first studies providing key evidence on the use of anthropometric indexes for predicting populations at high risk of MetS in resource-limited regions of China. Due to the variations in lifestyle patterns and access to health care services and optimal diet, the prevalence and manifestation of MetS in resource-limited areas can be different from those in more resource-abundant regions [5]. Findings from this study can serve as a benchmark for cross-regional comparisons. This evidence allows for a more nuanced development of regional health guidelines that are more attuned to local needs. By providing evidence for the most effective diagnostic indexes, this study also has the potential to support the development of scalable interventions that can be implemented in resource-limited settings.

### Limitations

Several limitations are present in this study. The interpretation of cause-and-effect relationships between anthropometric indexes and MetS was limited by the capture of data at a single time point in the cross-sectional design. The data were collected during the mid-to-late period of COVID-19, while the study was designed without predicting the impact of the pandemic on the population (eg, effects of altered lifestyle patterns resulting from the pandemic lockdown on body composition). Confounding factors that can only become apparent over time may be present. A lack of opportunity for collecting complete medical and medication histories due to ethical approval may represent a potential confounding factor. For example, the impact of a previous diagnosis of T2DM on the diagnostic performance of TyG is described in the discussion. Antihypertensive medication can potentially mask the presence of MetS in individuals who would be classified as at risk. However, more than 50% of the population at risk of hypertension was identified under the IDF criteria, suggesting that this limitation may have a limited impact on the overall conclusions. Besides, only IDF criteria were used to define MetS in this study. The harmonized definition of MetS is suggested to examine the consistency of our findings in future research. Since this study was based on residents living in China, the findings may have limited applicability to other ethnic groups.

### Conclusions

According to 8 anthropometric and lipid-related indices, it is suggested that WHtR and LAP are the most appropriate indexes for identifying the presence of MetS in resource-limited areas. Health care professionals are advised to factor in optimal thresholds of these anthropometric indexes from different regions when developing standardized criteria for disease diagnosis.

## Supplementary material

10.2196/57799 Multimedia Appendix 1 Supplementary materials regarding the study area, participant selection, discrimination of anthropometric indexes, and the relationship between the risk of metabolic syndrome and the anthropometric indexes.

## Abbreviations

ABSI: a body shape index
adjOR: adjusted odds ratio
AUC: area under the curve
CVD: cardiovascular disease
DBP: diastolic blood pressure
FPG: fasting plasma glucose
HbA_1c_: glycated hemoglobin
HDL-C: high-density lipoprotein cholesterol
IDF: International Diabetes Federation
LAP: lipid accumulation products
LDL-C: low-density lipoprotein cholesterol
MetS: metabolic syndrome
OR: odds ratio
RCS: restricted cubic spline
SBP: systolic blood pressure
TC: total cholesterol
TG: triglycerides
TyG: triglyceride-glucose
VAI: visceral obesity index
WC: waist circumference
WHtR: waist-to-height ratio
WWI: weight-adjusted waist index
