# Supplementary material for: Utility of Anthropometric Indexes for Detecting Metabolic Syndrome in Resource-Limited Regions of Northwestern China: Cross-Sectional Study
Source: JMIR Public Health Surveill. 2024 Nov 29;10:e57799. doi: 10.2196/57799 (PMC11622702; doi:10.2196/57799)
Supplement: Multimedia Appendix 1 [file publichealth-v10-e57799-s001.docx]

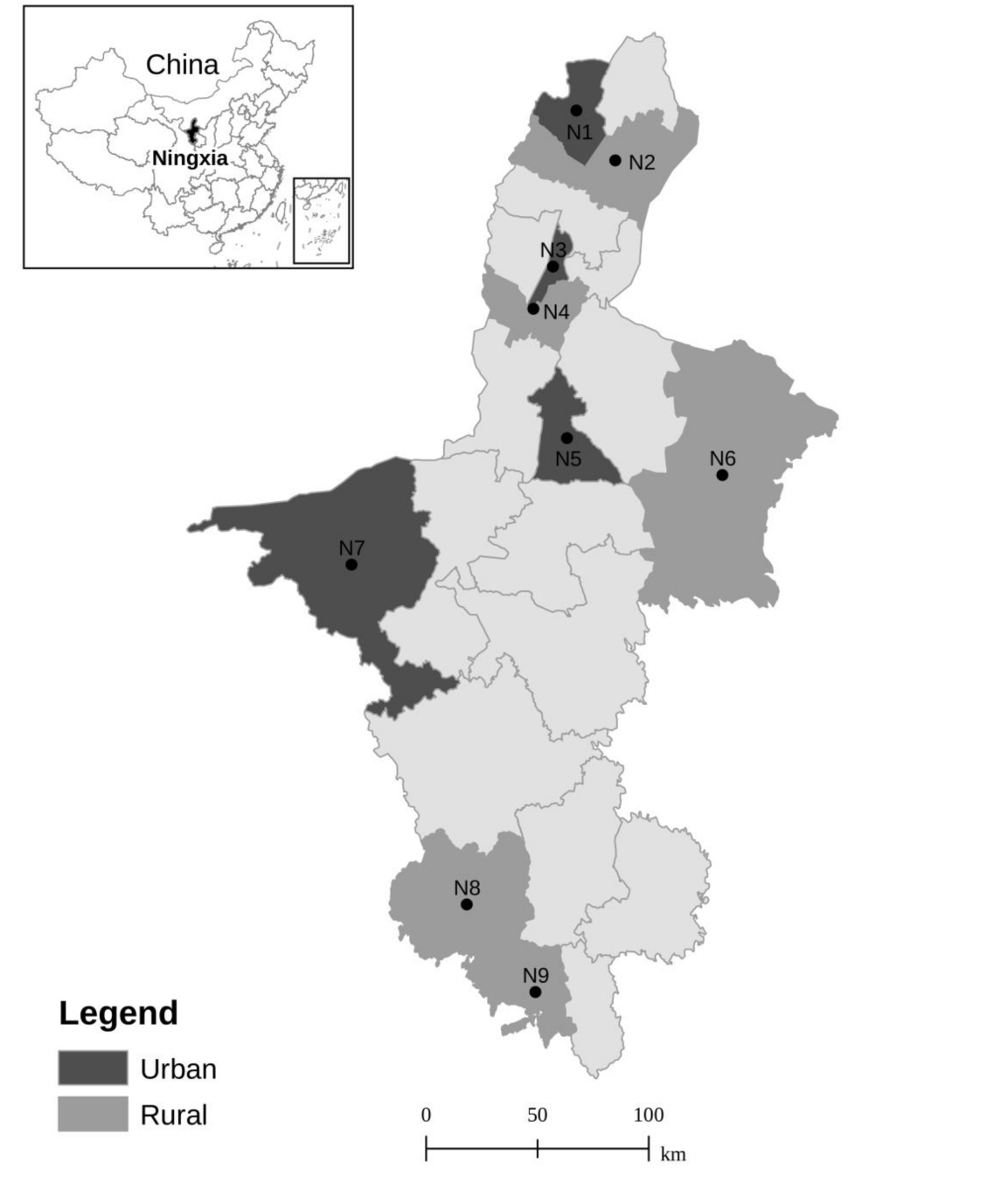


**Figure S1. Distribution of study area for cardiovascular disorders and the related risk factors survey in Ningxia Hui Autonomous Region**[1].


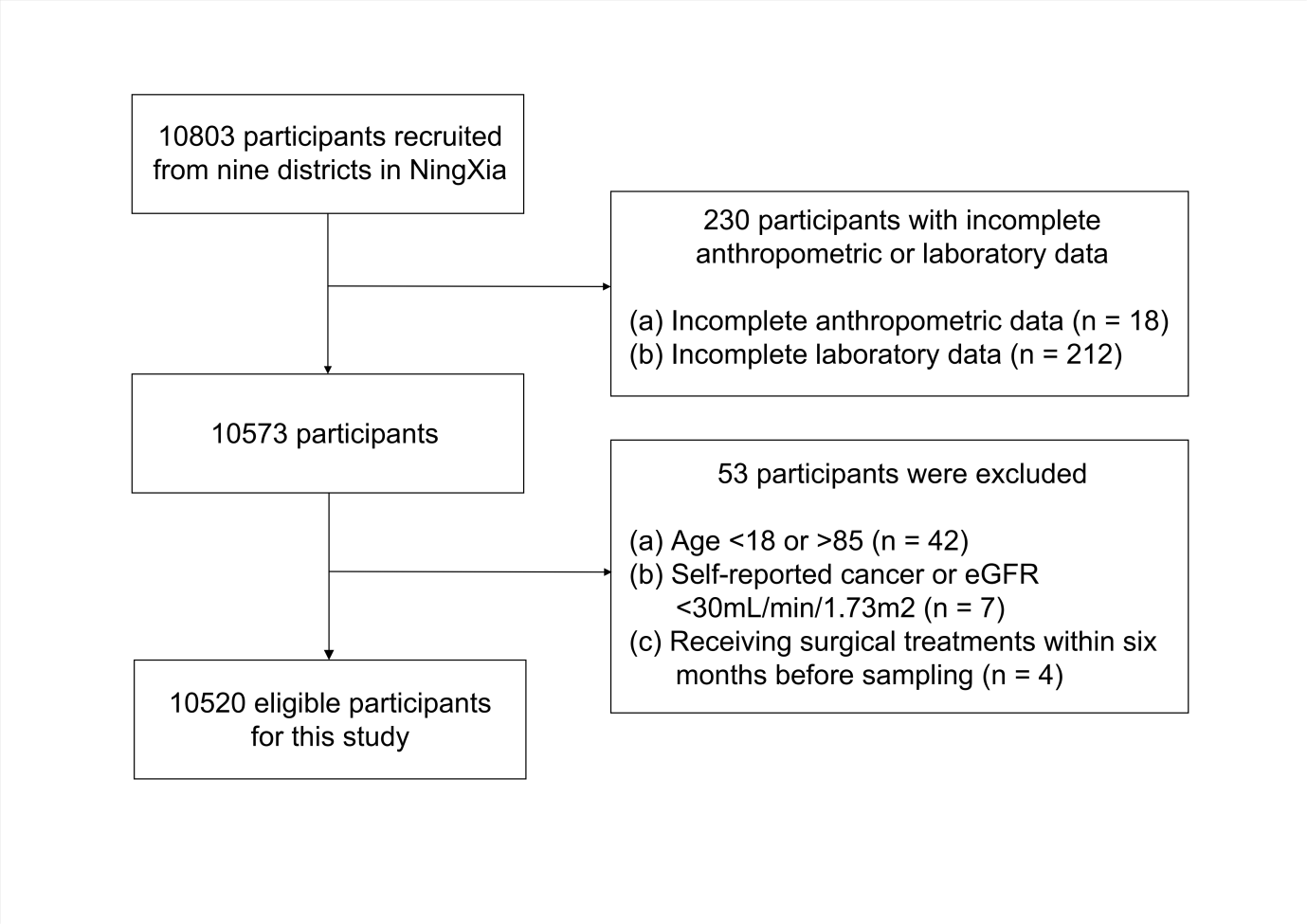


**Figure S2**. The flowchart of participant selection for this study.

| **Table S1**. Discrimination of eight anthropometric indexes for predicting metabolic syndrome, categorised by gender 10520 individuals from Ningxia Hui Autonomous Region, 2020-2021. | | | | | | |
| --- | --- | --- | --- | --- | --- | --- |
| Gender | Index | AUC (95% CI) | Cutoff | Sensitivity | Specificity | Youden Index |
| Female  (N=5679) | LAP a | 0.89 (0.89-0.90) | 28.67 | 0.84 | 0.77 | 0.61 |
|  | WHtR b | 0.88 (0.88-0.89) | 0.51 | 0.93 | 0.71 | 0.64 |
|  | VAI | 0.82 (0.81-0.84) | 1.76 | 0.76 | 0.74 | 0.51 |
|  | CI | 0.82 (0.81-0.83) | 1.2 | 0.79 | 0.71 | 0.5 |
|  | WWI | 0.81 (0.80-0.82) | 10.26 | 0.83 | 0.65 | 0.48 |
|  | BMI | 0.81 (0.80-0.82) | 24.71 | 0.81 | 0.67 | 0.49 |
|  | TyG Index | 0.81 (0.80-0.82) | 8.55 | 0.76 | 0.72 | 0.48 |
|  | ABSI | 0.75 (0.73-0.76) | 0.07 | 0.79 | 0.58 | 0.36 |
|  |  |  |  |  |  |  |
| Male  (N=4841) | WHtR a | 0.91 (0.90-0.92) | 0.53 | 0.88 | 0.8 | 0.69 |
|  | LAP b | 0.88 (0.87-0.89) | 40.23 | 0.76 | 0.83 | 0.59 |
|  | BMI | 0.85 (0.84-0.86) | 25.89 | 0.83 | 0.72 | 0.55 |
|  | CI | 0.83 (0.82-0.84) | 1.21 | 0.9 | 0.64 | 0.53 |
|  | WWI | 0.80 (0.79-0.82) | 10.29 | 0.79 | 0.68 | 0.47 |
|  | VAI | 0.78 (0.77-0.80) | 1.76 | 0.69 | 0.76 | 0.45 |
|  | TyG Index | 0.77 (0.76-0.79) | 8.85 | 0.69 | 0.75 | 0.44 |
|  | ABSI | 0.73 (0.72-0.75) | 0.08 | 0.66 | 0.69 | 0.35 |
| Abbreviations: BMI, body mass index; WHtR, waist-to-height ratio; WWI, weight-adjusted waist index; LAP, lipid accumulation products; ABSI, a body shape index; CI, conicity index; VAI, visceral obesity index; TyG Index, triglyceride-glucose index. | | | | | | |
| a The anthropometric indexe that showed the highest AUC values in each group. | | | | | | |
| b The anthropometric indexe that showed the second high AUC values in each group. | | | | | | |

**
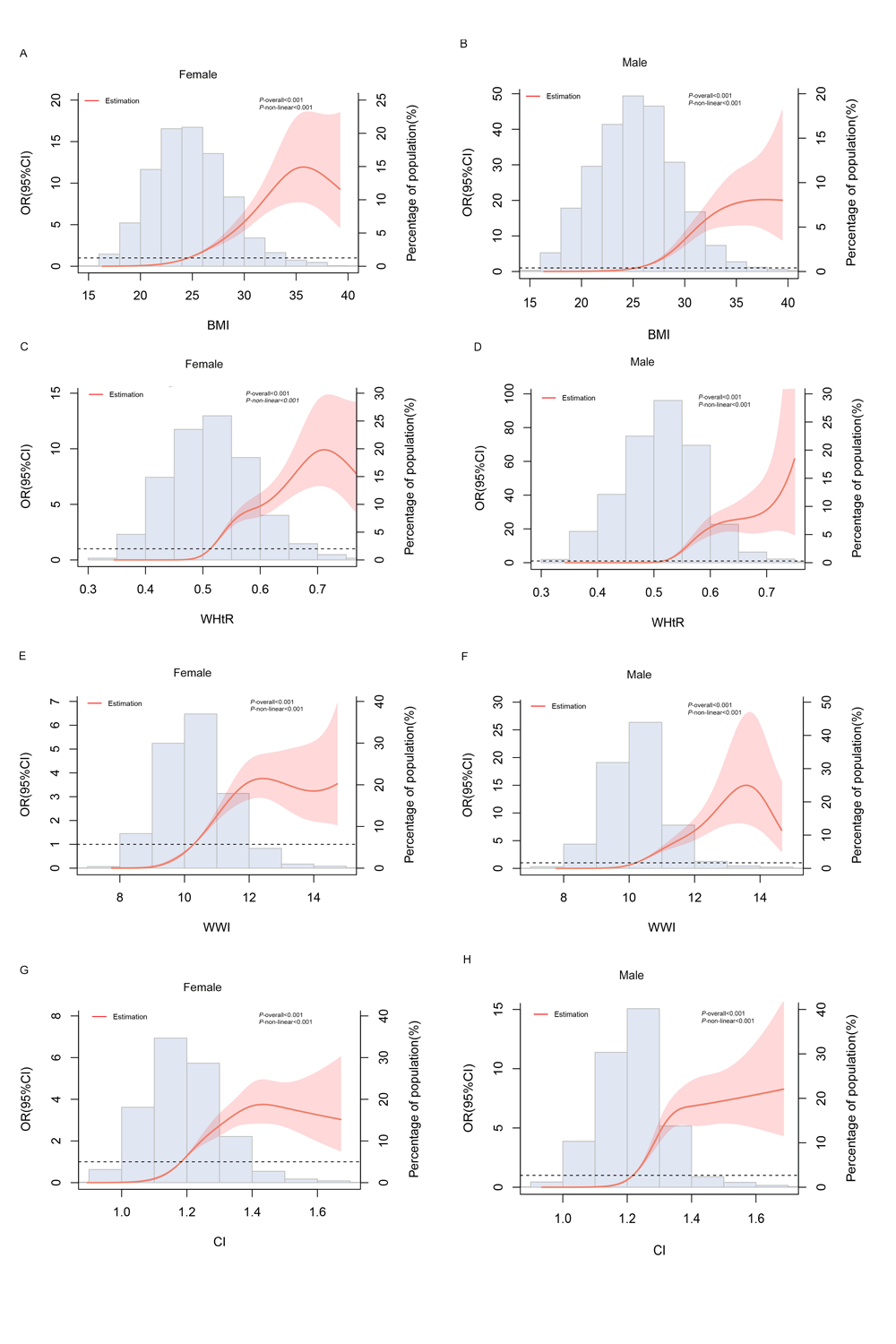
**

**
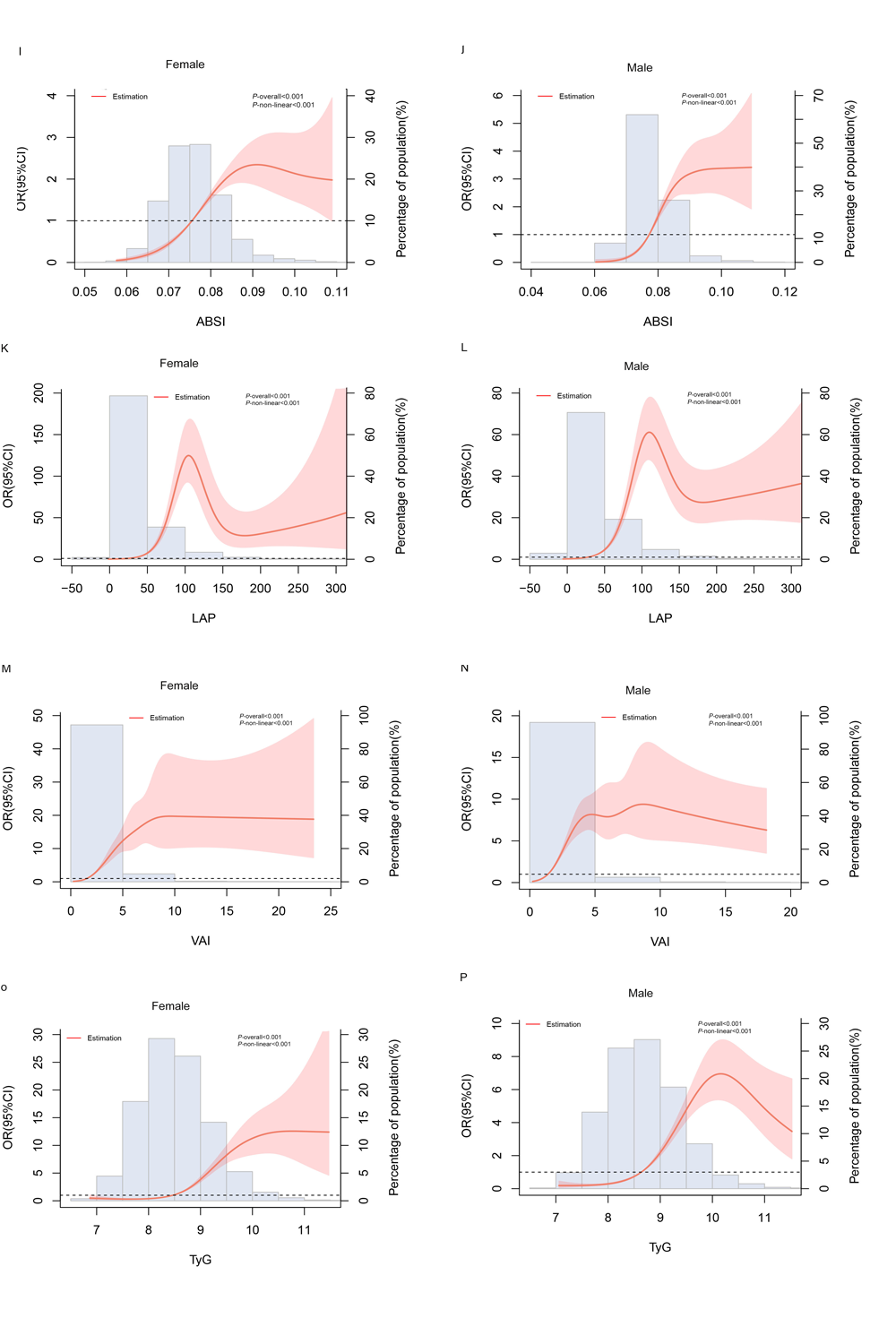
Figure S3.** Dose-response relationships between the risk of MetS and different anthropometric indexes in 10520 individuals from Ningxia Hui Autonomous Region, 2020-2021.

The red line represents the adjusted odds ratios (ORs) for the risk of metabolic syndrome across different anthropometric index values. The pink areas around the red lines represent the 95% confidence interval (CI) of these OR estimates. Bars represent the distribution of the study population across different values of these anthropometric indexes. Models were adjusted for age (years), ethnicity, current residence, education level, habitual alcohol consumption and tobacco use.

| **Table S2**. Discrimination of anthropometric indexes for predicting metabolic syndrome, categorised by age group in 10520 individuals from Ningxia Hui Autonomous Region, 2020-2021. | | | | | | |
| --- | --- | --- | --- | --- | --- | --- |
| Age group | Index | AUC (95% CI) | Cutoff | Sensitivity | Specificity | Youden Index |
| 18-34 years  (N=2877) | WHtR ^a^ | 0.93 (0.92-0.94) | 0.62 | 0.99 | 0.89 | 0.88 |
|  | LAP ^a^ | 0.93 (0.92-0.94) | 26.19 | 0.88 | 0.82 | 0.7 |
|  | BMI ^b^ | 0.91 (0.89-0.92) | 25.42 | 0.9 | 0.8 | 0.7 |
|  | VAI | 0.87 (0.85-0.89) | 1.58 | 0.83 | 0.77 | 0.6 |
|  | TyG Index | 0.84 (0.82-0.87) | 8.71 | 0.75 | 0.81 | 0.56 |
|  | CI | 0.83 (0.82-0.85) | 1.17 | 0.83 | 0.72 | 0.56 |
|  | WWI | 0.81 (0.79-0.83) | 9.88 | 0.78 | 0.72 | 0.5 |
|  | ABSI | 0.70 (0.67-0.73) | 0.07 | 0.77 | 0.55 | 0.32 |
|  |  |  |  |  |  |  |
| 35-64 years  (N=5712) | WHtR ^a^ | 0.86 (0.86-0.87) | 0.52 | 0.9 | 0.7 | 0.59 |
|  | LAP ^a^ | 0.86 (0.85-0.87) | 36.16 | 0.78 | 0.79 | 0.56 |
|  | BMI ^b^ | 0.81 (0.79-0.82) | 30.46 | 0.97 | 0.7 | 0.67 |
|  | VAI | 0.79 (0.78-0.80) | 1.2 | 0.7 | 0.77 | 0.46 |
|  | CI | 0.77 ( 0.76-0.79) | 1.21 | 0.78 | 0.64 | 0.42 |
|  | TyG Index | 0.77 (0.76-0.78) | 8.84 | 0.67 | 0.77 | 0.44 |
|  | WWI | 0.77 (0.75-0.78) | 10.27 | 0.79 | 0.61 | 0.4 |
|  | ABSI | 0.69 (0.67-0.70) | 0.08 | 0.69 | 0.59 | 0.28 |
|  | | | | | | |
| ≥65 years  (N=1931) | WHtR ^a^ | 0.86 (0.84-0.88) | 0.55 | 0.85 | 0.74 | 0.59 |
|  | LAP ^a^ | 0.86 (0.84-0.87) | 37.88 | 0.69 | 0.84 | 0.53 |
|  | BMI ^b^ | 0.78 (0.76-0.80) | 25.14 | 0.74 | 0.69 | 0.43 |
|  | VAI | 0.77 (0.75-0.79) | 1.76 | 0.68 | 0.75 | 0.43 |
|  | CI | 0.76 (0.74-0.79) | 1.46 | 0.98 | 0.53 | 0.51 |
|  | WWI | 0.76 (0.74-0.79) | 10.88 | 0.76 | 0.64 | 0.4 |
|  | TyG Index | 0.73 (0.71-0.76) | 8.83 | 0.58 | 0.79 | 0.37 |
|  | ABSI | 0.69 (0.66-0.71) | 0.08 | 0.77 | 0.52 | 0.29 |
| Abbreviations: Abbreviations: BMI, body mass index; BFP, body fat percentage; WHtR, waist to height ratio; WWI, weight-adjusted waist index; LAP, lipid accumulation products; ABSI, a body shape index; CI, conicity index; VAI, visceral obesity index; TyG Index, triglyceride-glucose index. | | | | | | |

| **Table S3.** Discrimination of anthropometric indexes for predicting metabolic syndrome, categorised by metabolic syndrome risk factors in 10520 individuals from Ningxia Hui Autonomous Region, 2020-2021.. | | | | | | |
| --- | --- | --- | --- | --- | --- | --- |
| Outcome | Index | AUC (95% CI) | Cutoff | Sensitivity | Specificity | Youden Index |
| Elevated TG  (N=2937) | TyG Index ^a^ | 0.98 (0.98-0.98) | 9.00 | 0.97 | 0.94 | 0.91 |
|  | VAI ^b^ | 0.97 (0.96-0.97) | 2.63 | 0.97 | 0.90 | 0.87 |
|  | LAP | 0.93 (0.93-0.94) | 55.33 | 0.97 | 0.88 | 0.86 |
|  | BMI | 0.69 (0.68-0.70) | 45.96 | 0.99 | 0.72 | 0.72 |
|  | WHtR | 0.67 (0.66-0.69) | 0.51 | 0.74 | 0.53 | 0.27 |
|  | CI | 0.63 (0.62-0.64) | 2.19 | 0.99 | 0.72 | 0.72 |
|  | WWI | 0.61 (0.60-0.62) | 9.83 | 0.81 | 0.38 | 0.19 |
|  | ABSI | 0.58 (0.57-0.60) | 0.15 | 0.99 | 0.72 | 0.72 |
|  | | | | | | |
| Elevated BP  (N=5350) | TyG Index | 0.68 (0.67-0.69) | 9.84 | 0.98 | 0.52 | 0.49 |
|  | VAI | 0.65 (0.64-0.66) | 7.97 | 0.99 | 0.5 | 0.49 |
|  | LAP ^b^ | 0.72 (0.71-0.73) | 75.29 | 0.95 | 0.56 | 0.51 |
|  | BMI | 0.70 (0.69-0.71) | 30.53 | 0.97 | 0.54 | 0.5 |
|  | WHtR ^a^ | 0.74 (0.73-0.75) | 0.58 | 0.93 | 0.59 | 0.51 |
|  | CI | 0.70 (0.69-0.71) | 1.56 | 0.99 | 0.5 | 0.49 |
|  | WWI | 0.70 (0.69-0.70) | 11.77 | 0.97 | 0.53 | 0.5 |
|  | ABSI | 0.65 (0.64-0.66) | 0.10 | 0.99 | 0.49 | 0.49 |
|  | | | | | | |
| Reduced HDL-C level  (N=4092) | TyG Index | 0.61 (0.60-0.62) | 8.52 | 0.62 | 0.55 | 0.17 |
|  | VAI ^a^ | 0.75 (0.74-0.76) | 1.56 | 0.70 | 0.68 | 0.38 |
|  | LAP ^b^ | 0.63 (0.62-0.64) | 306.36 | 0.99 | 0.61 | 0.61 |
|  | BMI | 0.59 (0.58-0.60) | 45.96 | 0.99 | 0.61 | 0.61 |
|  | WHtR | 0.60 (0.59-0.61) | 0.90 | 0.99 | 0.61 | 0.61 |
|  | CI | 0.54 (0.54-0.56) | 1.18 | 0.62 | 0.45 | 0.07 |
|  | WWI | 0.57 (0.56-0.58) | 9.91 | 0.70 | 0.40 | 0.10 |
|  | ABSI | 0.53 (0.51-0.54) | 0.08 | 0.51 | 0.52 | 0.04 |

| Table S3 continued. | | | | | | |
| --- | --- | --- | --- | --- | --- | --- |
| Outcome | Index | AUC (95% CI) | Cutoff | Sensitivity | Specificity | Youden Index |
| Raised FPG  (N=4382) | TyG Index ^a^ | 0.72 (0.71-0.73) | 8.65 | 0.63 | 0.69 | 0.32 |
|  |  |  |  |  |  |  |
|  | VAI | 0.63 (0.62-0.65) | 8.23 | 0.99 | 0.59 | 0.59 |
|  | LAP ^b^ | 0.66 (0.65-0.67) | 169.44 | 0.99 | 0.59 | 0.59 |
|  | BMI | 0.61 (0.60-0.62) | 45.96 | 0.99 | 0.58 | 0.58 |
|  | WHtR | 0.63 (0.63-0.64) | 0.85 | 0.58 | 0.58 | 0.58 |
|  | CI | 0.62 (0.60-0.63) | 1.88 | 0.99 | 0.58 | 0.58 |
|  | WWI | 0.61 (0.60-0.62) | 16.79 | 0.58 | 0.58 | 0.58 |
|  | ABSI | 0.59 (0.58-0.60) | 0.13 | 0.99 | 0.58 | 0.58 |
| Abbreviations: BMI, body mass index; WHtR, waist-to-height ratio; WWI, weight-adjusted waist index; LAP, lipid accumulation products; ABSI, a body shape index; CI, conicity index; VAI, visceral obesity index; TyG Index, triglyceride-glucose index.  ^a^ The anthropometric indexe that showed the highest AUC values in each group.  ^b^ The anthropometric indexe that showed the second high AUC values in each group. | | | | | | |

**Reference**

1. Shi H, Liu Y, Wang J, Luan H, Shi C. Prevalence of hyperuricaemia among adults from Ningxia Hui Autonomous Region, China: a cross-sectional study. BMJ Open. 2023 Oct 18;13(10):e072408. PMID: 37852763. doi: 10.1136/bmjopen-2023-072408.
